# An evaluation of the effects of caffeic acid phenethyl ester and Ankaferd blood stopper on secondary wound healing of oral mucosal tissue

**DOI:** 10.3906/sag-1908-114

**Published:** 2020-02-13

**Authors:** Mehmet GÜL, Ahmet GÜNAY, Abdulsamet TANIK

**Affiliations:** 1 Department of Periodontology, Faculty of Dentistry, Harran University, Şanlıurfa Turkey; 2 Department of Periodontology, Faculty of Dentistry, Dicle University, Diyarbakır Turkey; 3 Department of Periodontology, Faculty of Dentistry, Adıyaman University, Adıyaman Turkey

**Keywords:** Wound healing, vascular endothelial growth factor, tumour necrosis factor-inducible gene 6 protein

## Abstract

**Background/aim:**

Caffeic acid phenethyl ester (CAPE) and Ankaferd Blood Stopper (ABS) are considered to contribute to wound healing. The purpose of this study was to investigate the effect of ABS and CAPE on secondary wound healing of oral mucosal tissue.

**Materials and methods:**

In total, 63 male Sprague-Dawley rats were used in this study. The animals were randomly divided into three groups and anaesthetized with ketamine (8 mg/100 g, intraperitoneally): a control group, CAPE group, and ABS group. A full-thickness excisional wound was created using a 4 mm punch biopsy tool. Topical ABS and CAPE were then applied in each group for 7, 14, and 21 days (n = 7 in each group). The animals in each group were sacrificed after 7, 14, and 21 days. Palatal specimens were stained with haematoxylin-eosin. Vascular endothelial growth factor (VEGF) and tumour necrosis factor-inducible gene 6 (TSG-6) protein expressions were determined using the Western blot method.

**Results:**

Inflammation, vessel dilatation, and haemorrhages were significantly lower in the CAPE group as compared with these parameters in the other groups (P < 0.05). Fibrosis was significantly higher in the ABS group as compared with that in the other groups (P < 0.05). VEGF protein levels were elevated in the 21-day CAPE group and 7-day ABS group. The expression of TSG-6 increased in the 7-day CAPE group and 21-day ABS group.

**Conclusion:**

Based on our findings, ABS and CAPE had positive effects on the oral wound healing process.

## 1. Introduction

The wound healing process consists of four highly connected and overlapping processes: haemostasis, inflammation, proliferation, and tissue remodelling [1]. These stages and their biophysiological functions take place in a specific sequence, at a particular time, and for a specific period at an optimal intensity [2]. Many factors can affect wound healing and interfere with one or more phases of the wound healing process, leading to incomplete tissue repair [3]. After periodontal surgery, both bacterial contamination and plaque control affect the success of the surgery. Therapeutic agents are used to reduce bacterial plaque accumulation, prevent postoperative pain and tissue oedema, and accelerate wound healing [4].

Ankaferd Blood Stopper (ABS) is a plant extract that is widely used as a haemostatic agent in Turkey. ABS consists of *Glycyrrhiza glabra*,* Vitis vinifera*,* Alpinia officinarum*,* Urtica dioica*, and* Thymus vulgaris* [5]. Previous research demonstrated the effectiveness of ABS in bleeding control, as well as its strong antimicrobial properties [6]. According to a previous study, the haemostatic activity and antimicrobial properties of ABS suggest that it may be useful in dental treatments [7].

Caffeic acid phenethyl ester (CAPE) is an active component of propolis, which is a compound produced by bees from substances that the bees collect from plants. Propolis-based products are used in the cosmetic industry, as well as in the therapeutic field, primarily for their antibacterial and antiinflammatory effects, and the therapeutic properties of propolis are well accepted [8].

Considering the reported beneficial effects of ABS and CAPE, it is reasonable to speculate that they might accelerate the healing process of mucosal wounds [7,8]. Therefore, the aim of the present study was to investigate the effects of topical ABS and CAPE on secondary healing of the surfaces of experimental excisional wound areas created in the palatal mucoperiosteum of rats.

## 2. Materials and methods

Sixty-three male Sprague-Dawley rats (mean age: 7 weeks; weight: 280–490 g) were used in this study. The study was conducted at the Health Institution Research Centre, Dicle University, Diyarbakır, Turkey (ethics committee approval no: 2015/17). 

The rats were housed individually in plastic cages in a controlled environment (21 °C and a 12 h light/12 h dark cycle), with free access to drinking water and food. They were randomly divided into three groups and anaesthetized with ketamine (8 mg/100 g, intraperitoneally): control group, CAPE group, and ABS group. Palatal mucosal defects were created in the three groups. The rats in the ABS group were topically treated with 0.10 mL of ABS solution, and the rats in the CAPE group were topically treated with 100 mmol/kg of CAPE. Pressure was applied to a sterile pad until bleeding control was achieved. All treatments were applied for 7, 14, and 21 days (n = 7).

### 2.1. Creation of palatal mucosal defects

Prior to the creation of palatal mucosal defects (4 mm), intramuscular ketamine HCl (Alfamine 10%; Alfasan, Woerden, the Netherlands) at a dose of 45 mg/kg and xylazine HCl (Alfazyne 2%; Alfasan) at a dose of 2.50 mg/kg were administered. The rats were prepared for the surgical procedure in accordance with standard disinfection and sterilization guidelines, and the surgery was performed using sterile instruments. A 4-mm punch biopsy instrument was used to create a full-thickness excisional wound area between the molar teeth. 

After the surgical procedure, the wound areas were allowed to recover (i.e., secondary healing). All the rats in each group were sacrificed by a single dose of sodium thiopentone (lethal dose: 60 mg/kg) administered intraperitoneally. Palatal tissue specimens, including the tissue around the wound site area, were obtained. The tissues were stored in 10% formaldehyde for histological evaluation. For biochemical analysis, the tissues taken with the punch biopsy were stored in Eppendorf tubes at –80 °C until the time of the analysis. 

### 2.2. Histopathological method

The palatal specimens were fixed directly in neutral buffered formalin fixative. After complete fixation of the tissues, they were washed under running water for 12 h. The specimens were dehydrated through a graded alcohol series (30%, 50%, 70%, 80%, 90%, 96%, and 100%) for 12 h, followed by immersion in xylene, infiltration, and embedding in paraffin blocks. Sections of paraffin-embedded tissues 5 µm thick were stained with haematoxylin-eosin dye and placed on poly-L-lysine-coated slides. They were placed in an incubator at 60 °C overnight. After the sections had cooled, they were cleared in xylene three times for 2–5 min, followed by passing through 96%, 80%, 70%, and 60% ethyl alcohol for 5 min. They were placed in distilled water for 5 min. The sections were placed in EDTA solution to dissolve the palatal tissue. The sections were mounted on slides. They were placed in citric acid (pH 6) for 7 + 5 min to remove antigen masking. They were cooled for 20 min at room temperature, washed with phosphate-buffered saline (PBS) solution (3 × 5 min), and placed in 3% hydrogen peroxide for 20 min for endogenous peroxide blockade. The sections were washed again with PBS (3 × 5 min) and placed in an incubation container. All subsequent procedures were carried out in this incubation container. 

Vascular endothelial growth factor (VEGF) (mouse monoclonal, sc-7269, Santa Cruz Biotechnology, USA) and tumour necrosis factor-inducible gene 6 (TSG-6) (mouse monoclonal, sc-65886, Santa Cruz Biotechnology) antibodies were applied to the sections. The primary antibody was applied for 1 h and washed with PBS solution (3 × 5 min). The secondary antibody (Zymed and Histostain-Plus Kit; Invitrogen) was marked with streptavidin and dripped and left for 30 min in the closed moist box, followed by washing with PBS solution (3 × 5 min). Aminoethyl carbazole was used as a chromogen. This step was followed by washing with distilled water to prevent the reaction of the antigen/antibody. Following contrast staining with Mayer’s haematoxylin, the samples were washed in distilled water again. In the final stage, for immunohistopathological analysis, the sections were evaluated using a photomicroscope (Nikon Eclipse i50; Nikon, Tokyo, Japan) for blind evaluation.

### 2.3. Western blotting method 

#### 2.3.1. Cell lysis and protein quantitation

The palatal tissue was frozen in liquid nitrogen and powdered in a porcelain mortar. The powdered placental tissue (50 mg) was placed in 250 μL of RIPA lysis solution containing a protease inhibitor mixture in ice for 1 h. The lysed placental samples were stored at –86 °C. All the steps were performed on ice to prevent protein degradation. The total cellular protein concentration was calculated using a Pierce BCA Kit (Thermo Scientific, Waltham, MA, USA).

#### 2.3.2. Sodium dodecyl sulphate-polyacrylamide gel electrophoresis (SDS-PAGE)

The protein samples were prepared in SDS loading solution (2% SDS, 5% glycerol, 0.01% bromophenol blue, and 8% DL-dithiothreitol) and boiled at 95 °C for 5 min. The protein samples (20 μg) were loaded into a 10% polyacrylamide gel, and electrophoresis was performed using buffer solution (2.40 mm Tris, 19.20 mm glycine, and 0.01% SDS) for 1 h at 200 V.

#### 2.3.3. Membrane transfer and antibody staining of proteins

The separated proteins were transferred from SDS-PAGE to a PVDF membrane at 100 V. The membrane was blocked at room temperature for 1 h and 5% milk powder prepared in PBS solution. The membrane was treated with primary antibodies at room temperature for 2 h, followed by washing four times with PBS-T for 30 min. Subsequently, the membrane was treated with horseradish peroxidase-conjugated secondary antibodies at room temperature for 1 h at a 1:10,000 dilution rate. Finally, the membrane was washed again four times with PBS for 30 min. Protein bands were visualized using an enhanced chemiluminescent reagent chemical (Bio-Rad, Hercules, CA, USA). 

### 2.4. Statistical analysis

The power of the study was calculated as 80% on the epithelial regeneration of the palatal oral mucosa of rats used in the experiment. The effect size was 0.5 and the α value was taken as 0.05. Sample size estimation was performed by using G*Power (Franz Faul, University of Kiel, Kiel, Germany) version 3.1.9.2. Statistical analysis of the data was performed using the SPSS version 21.0 for Windows (IBM Corp., Armonk, NY, USA) statistical program. The histological data and numerical values are shown as mean (mean) and standard deviation. The Mann–Whitney U test was used for comparisons of two groups. A Bonferroni-corrected Mann–Whitney U test was used for comparisons of more than two groups. In all statistical tests, a value of P < 0.05 was considered significant.

## 3. Results

### 3.1. Histopathological findings

**Figure 1:**
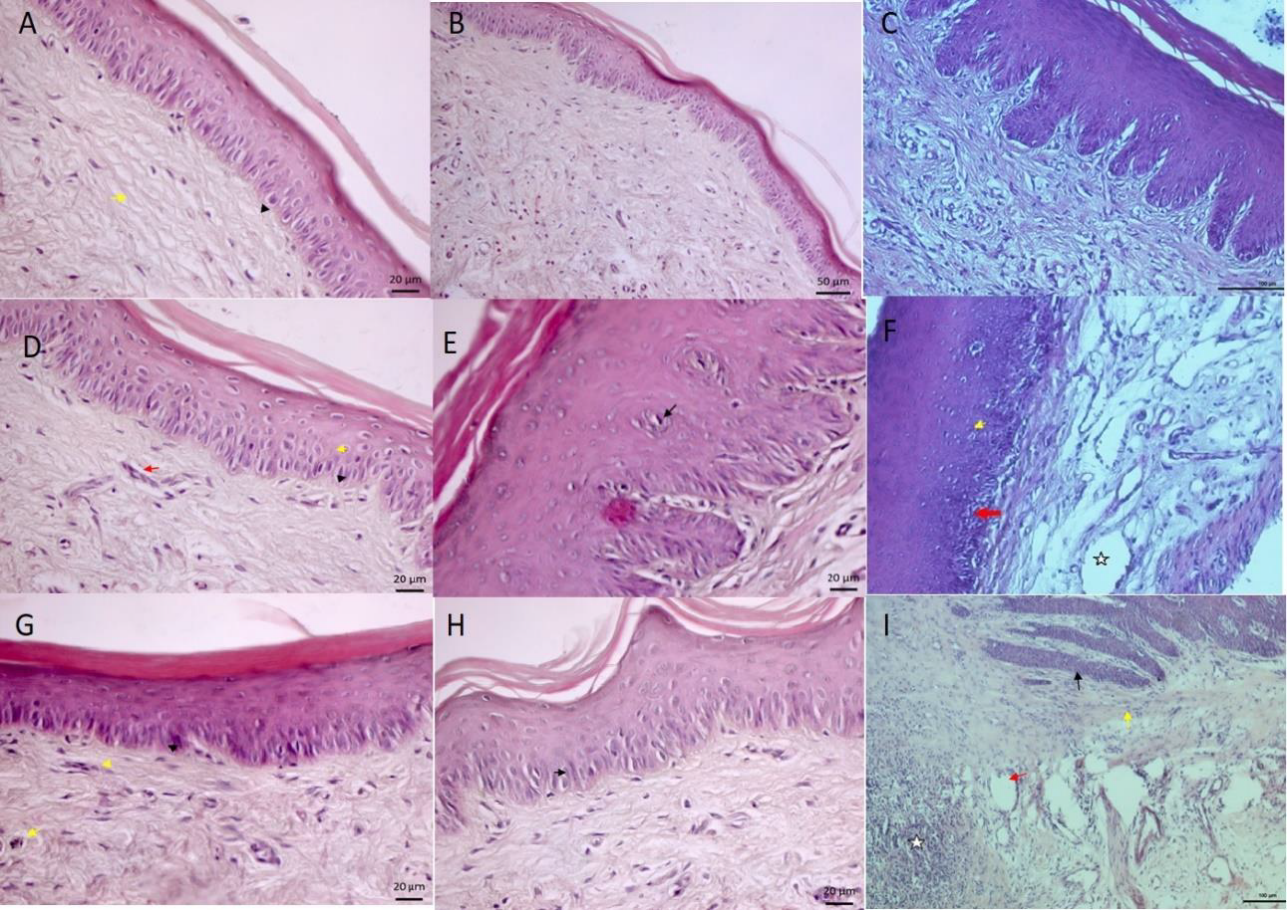
A) 7th day ABS group; haematoxylin-eosin staining bar 20 μm. B) 7th day CAPE group; haematoxylin-eosin staining bar 50 μm. C) 7th day control group; haematoxylin-eosin staining bar 100 μm. D) 14th day ABS group; haematoxylin-eosin staining bar 20 μm. E) 14th day CAPE group; haematoxylin-eosin staining bar 20 μm. F) 14th day control group; haematoxylin-eosin staining bar 100 μm. G) 21st day ABS group; haematoxylin-eosin staining bar 20 μm. H) 21st day CAPE group; haematoxylin-eosin staining bar 20 μm. I) 21st day control group; haematoxylin-eosin staining bar 100 μm.

In the 7-day ABS group, small vacuous structures were observed, with mild hypertrophy of basal cells. Connective tissue and collagen fibres were clearly observed (Figure 1A). In the 7-day CAPE group, mitotic activity in the germinal epithelium increased, and mononuclear cells were individually scattered in connective tissue (Figure 1B). In the 7-day control group, the basal cells were prismatic in appearance, and collagen fibres were arranged in a parallel fashion in connective tissue. In addition, connective tissue cells were distributed freely, and the vascular structure appeared normal (Figure 1C).

The 14-day ABS group was characterized by hyperplasia in the basal cells, in addition to hypertrophy of spinosum cells. Small clusters of connective tissue and mononuclear cell infiltration were also observed (Figure 1D). In the 14-day CAPE group, numerous cylindrical cells were observed in the basal layer, and secondary papillary structures were detected in the epithelium. Collagen fibres and vascular structure were also visible (Figure 1E). In the 14-day control group, the basal cells were prismatic in appearance, and collagen fibres of connective tissue were arranged in a parallel fashion. Connective tissue cells were scattered freely, and the vascular structure appeared normal (Figure 1F). 

In the 21-d ABS group, hyperplasia was present in the basal cells, and small groups of mononuclear cells were visible in connective tissue (Figure 1G). In the 21-day CAPE group, there was an increase in mitotic activity in the germinal epithelium and inflammatory cells in some connective tissue areas (Figure 1H). In the control group, the basal cells were prismatic in appearance, and collagen fibres were arranged in a parallel manner in connective tissue. In addition, connective tissue cells were freely distributed, and the vascular structure appeared normal (Figure 1I). 

### 3.2. Immunohistochemical findings of TSG-6

**Figure 2:**
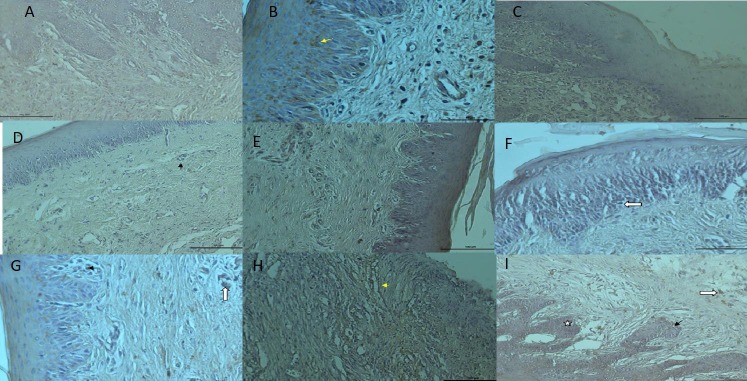
A) 7th day ABS group; TSG-6 immunostaining bar 100 μm. B) 7th day CAPE group; TSG-6 immunostaining bar 50 μm. C) 7th day control group; TSG-6 immunostaining bar 100 μm. D) 14th day ABS group; TSG-6 immunostaining bar 100 μm. E) 14th day CAPE group; TSG-6 immunostaining bar 100 μm. F) 14th day control group; TSG-6 immunostaining bar 100 μm. G) 21st day ABS group; TSG-6 immunostaining bar 50 μm. H) 21st day CAPE group; TSG-6 immunostaining bar 100 μm. I) 21st day control group; TSG-6 immunostaining bar 100 μm.

In the 7-day ABS group, a TSG-6-positive reaction was observed in small numbers of inflammatory cells in the small vessels of connective tissue (Figure 2A). In the 7-day CAPE group, TSG-6 expression was positive in polygonal cells in the spinosum layer, and the TSG-6 reaction was positive in a few cells in connective tissue (Figure 2B). In the 7-day control group, TSG-6 was not expressed in epithelial cells, and weak TSG-6 expression was detected in cells in the papillary region and deep connective tissue areas (Figure 2C).

In the 14-day ABS group, TSG-6-positive cells were observed in small vessels in connective tissue and in a few inflammatory cells (Figure 2D). TSG-6-positive cells were also detected in some basal cells in the 14-day CAPE group and in a small number of vascular cells (Figure 2E). In the 14-day control group, TSG-6 was not expressed in epithelial cells, and weak TSG-6 expression was observed in cells in the papillary region and deep connective tissue areas (Figure 2F).

In the 21-day ABS group, TSG-6-positive expression was observed in a small number of cells in the papillary area and macrophages in connective tissue (Figure 2G). TSG-6-positive expression was also observed in the basal membrane structure of blood vessels in the 21-day CAPE group (Figure 2H). In the 21-day control group, TSG-6 was not expressed in epithelial cells, and weakly expressed in cells in the papillary region and deep connective tissue (Figure 2I). 

### 3.3. Immunohistochemical findings of VEGF

**Figure 3:**
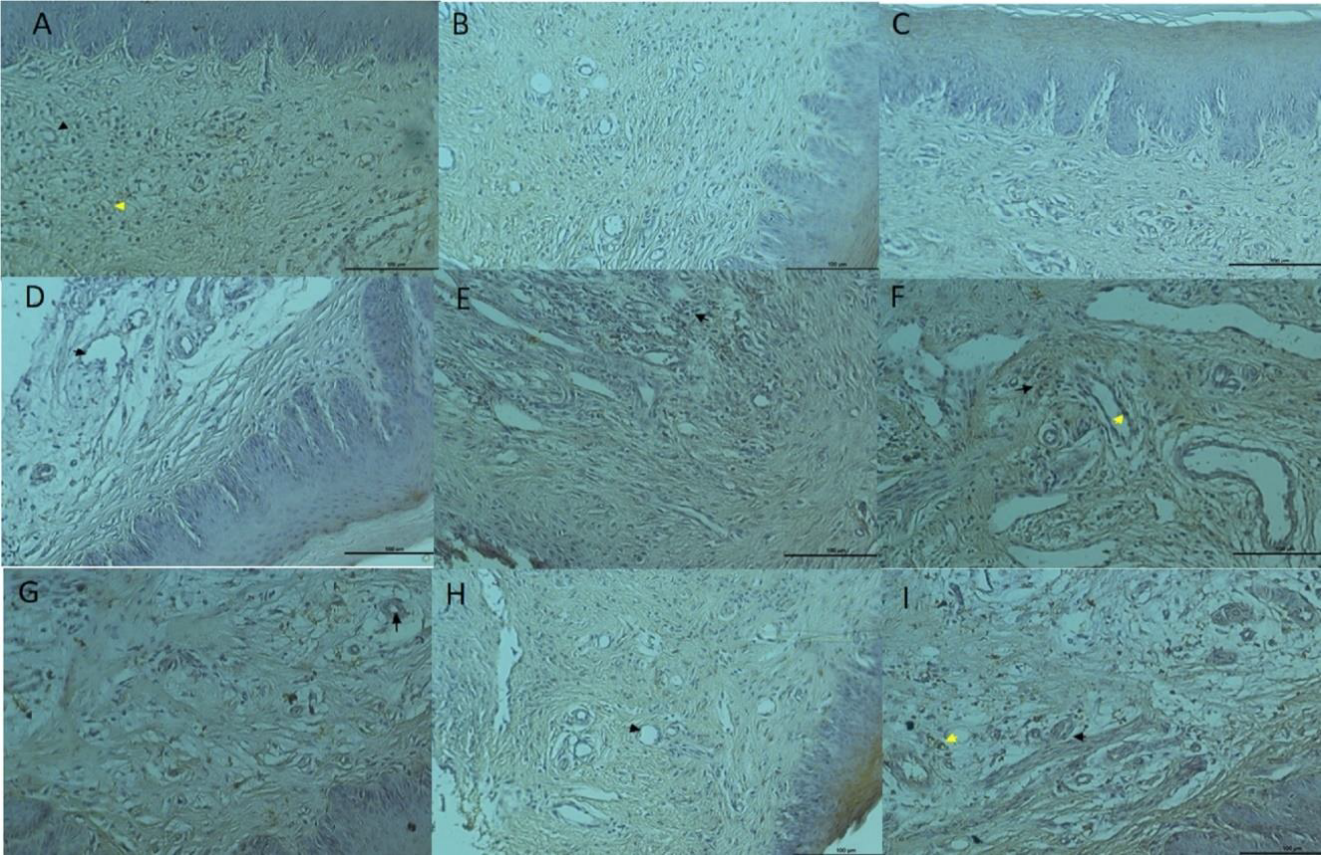
A) 7th day ABS group; VEGF immunostaining bar 100 μm. B) 7th day CAPE group; VEGF immunostaining bar 100 μm. C) 7th day control group; VEGF immunostaining bar 100 μm. D) 14th day ABS group; VEGF immunostaining bar 100 μm. E) 14th day CAPE group; VEGF immunostaining bar 100 μm. F) 14th day control group; VEGF immunostaining bar 100 μm. G) 21st day ABS group; VEGF immunostaining bar 100 μm. H) 21st day CAPE group; VEGF immunostaining bar 100 μm. I) 21st day control group; VEGF immunostaining bar 100 μm.

In the 7-day ABS group, a VEGF-positive reaction was observed in diffuse connective tissue cells in connective tissue areas, in addition to weak expression in vascular endothelial cells (Figure 3A). VEGF was not expressed in blood vessels and connective tissue in the 7-day CAPE group (Figure 3B). Weak VEGF expression was observed in vascular endothelial cells and connective cells in the 7-day control group (Figure 3C).

In the 14-day ABS group, VEGF was not expressed in endothelial cells in vessel walls (Figure 3D). A VEGF-positive reaction was observed in a small number of inflammatory cells around the vessels in the 14-d CAPE group (Figure 3E). Poor VEGF expression was observed in vascular endothelial cells and connective cells in the 14-d control group (Figure 3F).

In the 21-day ABS group, VEGF was not expressed in endothelial cells in blood vessels, whereas a VEGF-positive reaction was observed in mononuclear cells, which were sparsely distributed in connective tissue (Figure 3G). VEGF expression was not detected in blood vessels and connective tissue of the 21-day CAPE group (Figure 3H). Poor VEGF expression was observed in vascular endothelial cells and connective cells in the 21-day control group (Figure 3I).

### 3.4. Results of the statistical analysis of the three groups 

We evaluated whether epithelium regeneration, fibrosis, inflammation, vascular dilatation, and haemorrhages had a significant effect on wound healing, with these parameters assigned values between 0 and 4. 

Tables 1, 2, and 3 present data on the mean of the histopathological values of the rats on days 7, 14, and 21, respectively. The tables show the mean values for epithelium regeneration, fibrosis, inflammation, vascular dilatation, and haemorrhages in the groups, in addition to the findings in the ABS group versus the control group, control group versus the CAPE group, and ABS group versus the CAPE group. As shown in Table 1, inflammation decreased significantly in the CAPE group versus the control group and in the ABS group versus the CAPE group (P < 0.05). In addition, vascular dilatation and haemorrhages decreased significantly in the ABS and CAPE groups as compared with these parameters in the control group (P < 0.05). As presented in Table 2, fibrosis increased significantly in the control group as compared with that in the ABS group. In the comparison between ABS group and CAPE group, fibrosis decreased significantly (P < 0.05). There was no statistically significant difference in any other parameters between the other groups. As shown in Table 3, epithelial regeneration decreased significantly in the ABS group versus the CAPE group (P < 0.05). Vascular dilatation and haemorrhages increased significantly in the control group as compared with that in the CAPE group (P < 0.05). There were no statistically significant differences in any other parameters between the groups. 

**Table 1:** Comparison of histopathological values at day 7.

	Controln = 7	ABSn = 7	CAPEn = 7	Kruskal–Wallis P-value	Mann–WhitneyU P-value
Epithelium regeneration	0.86 ± 0.69	1.43 ± 0.54	0.57 ± 0.54	0.052	NS*, NS** 0.018***
Fibrosis	0.57 ± 0.54	0.86 ± 0.69	1.00 ± 0.58	0.405	NS*, NS** NS***
Inflammation	0.43 ± 0.54	0.71 ± 0.49	0.43 ± 0.54	0.483	NS*, NS** NS***
Vascular dilatation and haemorrhage	0.57 ± 0.54	1.00 ± 0.58	1.29 ± 0.49	0.073	NS*, 0.030** NS***

*ABS group comparison with control group (P < 0.05); **Comparison of control group with CAPE group (P < 0.05); ***Comparison of ABS group with CAPE group (P < 0.05), NS: Not significant.

**Table 2:** Comparison of histopathological values at day 14.

	Controln = 7	ABSn = 7	CAPEn = 7	Kruskal–Wallis P-value	Mann–WhitneyU P- value
Epithelium regeneration	0.57 ± 0.53	0.86 ± 0.69	0.71 ± 0.49	0.689	NS*, NS**, NS***
Fibrosis	1 ± 0.58	0.71 ± 0.76	1 ± 0.58	0.585	NS*, NS**, NS***
Inflammation	1.29 ± 0.76	1.29 ± 0.76	0.29 ± 0.49	0.027	NS*, 0.020**, 0.020***
Vascular dilatation and haemorrhage	1.43 ± 0.54	0.71 ± 0.49	0.71 ± 0.49	0.035	0.030*, 0.030** NS***

*ABS group comparison with control group (P < 0.05), **Comparison of control group with CAPE group (P < 0.05), ***Comparison of ABS group with CAPE group (P < 0.05), NS: Not significant.

**Table 3:** Comparison of histopathological values at day 21.

	Controln = 7	ABSn = 7	CAPEn = 7	Kruskal–Wallis P- value	Mann–WhitneyU P- value
Epithelium regeneration	1.00 ± 0.58	1.29 ± 0.76	0.57 ± 0.54	0.136	NS*, NS** NS***
Fibrosis	0.57 ± 0.54	2.71 ± 0,49	1.14 ± 0.69	0.001	0.001*, NS** 0.003***
Inflammation	1.14 ± 0.69	1.14 ± 0.69	0.57 ± 0.54	0.184	NS*, NS** NS***
Vascular dilatation and haemorrhage	0.86 ± 0.69	0.86 ± 0.69	0.71 ± 0.49	0.910	NS*, NS** NS***

*ABS group comparison with control group (P < 0.05), **Comparison of control group with CAPE group (P < 0.05), ***Comparison of ABS group with CAPE group (P < 0.05), NS: Not significant.

### 3.5. Results of the biochemical analysis

**Figure 4:**
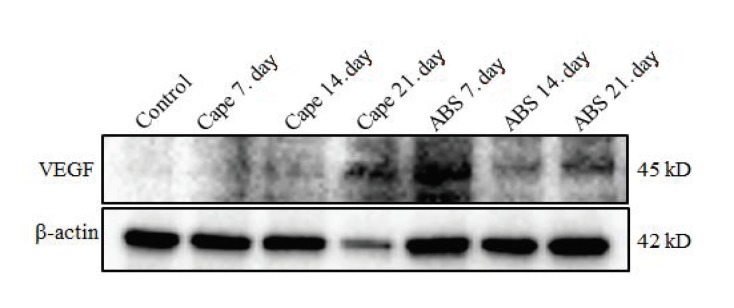
Twenty μg total protein gel was run. Anti-VEGF and anti-β-actin antibodies were analysed using the western blotting method. β-actin was used as loading control.

**Figure 5:**
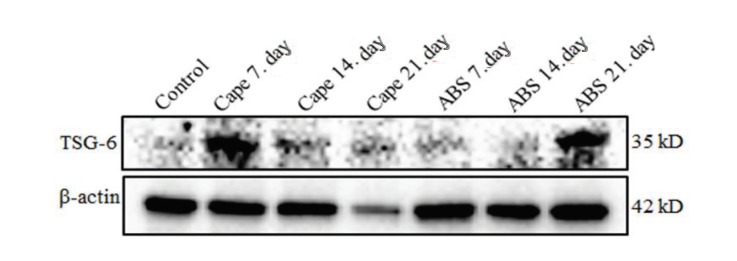
Twenty μg total protein gel was run. Anti-TSG-6 and anti-β-actin antibodies were analysed using the western blotting method. β-actin was used as loading control.

In the 7-day ABS group and 21-day CAPE group, the expression of VEGF in palatal tissue increased dramatically (Figure 4). TSG-6 expression in palatal tissue also markedly increased in the 7-day CAPE group and 21-day ABS group (Figure 5).

## 4. Discussion 

Many factors have an adverse effect on wound healing at various stages of the wound healing process, thereby impairing tissue production and impeding wound healing [9]. 

In a previous study, Kosger et al. induced 10 mm full-thickness palatal mucosal wounds in rats and then applied *Arnebia densiflora* extract topically to the surface of the wounds. The rats were sacrificed 4, 7, 14, and 21 days later, and palatal tissue specimens were evaluated histologically [10]. Tramontina et al. investigated the effect of bismuth subgallate on wound healing in rats in which a 3.50 × 2 mm wound was created. They performed a histological and morphological analysis 1, 4, 7, 11, and 18 days later [11]. Kozlovsky et al. studied the effects of various antimicrobial agents on wound healing of a soft tissue defect with a diameter of 5 mm in the palatal mucosa of rats [12]. Yamashita et al. used the rat palatal space as an experimental wound healing model to assess the impact of zoledronate on bone and mucosal wounds [13]. Koluman et al. suggested that the concentrations of the antioxidants and other specific molecules in ABS have not been affected after exposure to the synthetic gastric fluid. Those findings have provided the basis for future ABS clinical trials that will be designed for the oral-systemic administration to control haemorrhages of the gastrointestinal system tract [14]. Sezgin et al. excisional wounds with a diameter of 3 mm were made in the centre of the palatal mucosa and they determined the number of subjects of each group to be 7 [15]. Ersel et al. evaluated in vivo wound healing effects of a sericin-containing gel formulation in an incision wound model in rats and they determined the number of subjects of each group to be 7 [16].

In the present study, we created an excisional wound model in rat palatal mucosa and then evaluated secondary healing. We determined the number of subjects of each group to be 7. In our study, tissue samples were obtained 7, 14, and 21 days after CAPE and ABS treatment to evaluate the effects of CAPE and ABS on wound healing in oral mucosal tissue of rats. Before the study, a preliminary evaluation was undertaken to determine the ideal defect width for histological and biochemical evaluations. Based on this evaluation and to standardize the excisional surgery, a full-thickness defect with a diameter of 4 mm was created by punch biopsy.

According to Demiralp et al., ABS inhibited the growth of bacteria, as well as haemostatic activity. Given the antiinfective effect of ABS, it may be useful in the clinical setting, for example, in the treatment of traumatic wounds. Thus, it is important to determine its effectiveness against various pathogens [17]. In the present study, vessel dilatation and haemorrhages decreased in the 7-day ABS group. This finding suggested that in the early stage of wound healing, ABS may reduce bleeding by forming a protein network and consequently decrease the side effects of bacterial infection in a wound. It may also reduce inflammation, thereby exerting a positive effect on wound healing.

A recent study on ABS-treated defects observed a reduction in wound width and an increase in wound contraction. The authors attributed these findings to an increase in the rate of healing and a reduction in inflammation due to the antioxidant activity of the components in ABS [18]. Yüce et al. suggested that on days 5–10, the inflammatory granulation tissue in the ABS-treated wounds was less than in the normal wounds. By day 30, all of the wounds had achieved the same symptomatic state [19]. In the present study, we observed no positive effects of ABS on wound inflammation at 7, 14, or 21-days.

Reepithelization occurs in the proliferative phase of wound healing due to stimulation of growth factors, which are synthesized and secreted by platelets and macrophages [20]. Epithelial cells, which extend toward the edge of a wound, are the basic elements of wound healing, starting from the edge of the wound as a result of migration from the central membrane toward the base of the wound [21]. In the present study, increased epithelial regeneration, with positive effects on wound healing, was observed in the 7, 14, and 21-day ABS groups, although the increase in epithelial regeneration was not statistically significant. 

In a rat study, Ögetürk et al. created soft tissue damage using carbon tetrachloride (CCl4) and applied CAPE to the wound. They observed no pathology in a control group, but the epithelium was reduced and pulmonary interstitium haemorrhages were detected in the CCl4 group. In addition, they observed polymorphous core leukocytes and macrophage infiltration. In the CCl4 CAPE-treated group, they observed mild haemorrhage areas similar to the control group [22]. 

In the present study, vessel dilatation and haemorrhages were decreased in the 7-day CAPE group. We speculate that CAPE may exert a positive effect on wound healing by decreasing bleeding and inflammation. In the 14-day CAPE group, vessel dilatation and haemorrhages decreased, but the reduction was not statistically significant. In the 21-day CAPE group, vessel dilatation and haemorrhages increased significantly as compared with these parameters in the control group. These findings may be attributed to the loss of antiinflammatory and haemostatic activity of CAPE in the later wound healing period. 

Previous research reported that CAPE exhibited strong antioxidant, antiinflammatory, and wound healing properties, with the latter attributed to the effect of CAPE on nuclear factor-κB inhibition [23]. In previous in vitro and in vivo studies, CAPE was applied at micromolar concentrations. During inflammation, arachidonic acid showed various biological activities, such as suppression of the lipoxygenase pathway and inhibition of nuclear factor-κB [24]. Magro-Filho and De Carvalho demonstrated that topical applications of CAPE to wound surfaces in oral cavity surgery reduced inflammation and had an analgesic effect [25]. In the present study, inflammation decreased significantly in the 7-day CAPE group. Although inflammation also improved in the 14-day CAPE group, the improvement was not statistically significant. There was no reduction in inflammation in the other groups. The positive effects of CAPE on wound healing may be due to its antiinflammatory effects and antimicrobial properties. Özyurt et al. reported that CAPE was superior to E-vit in preventing bleomycin-induced lung fibrosis. CAPE also reduced hydroxyproline, malondialdehyde (MDA), and myeloperoxidase (MPO) levels as compared with those in a control group, although the E-vit treatment was most successful [26].

In the present study, fibrosis decreased in rats in the 7-day CAPE group, although this finding was not statistically significant as compared with that in the other groups. Fibrous tissue is undesirable in wound healing. We think that CAPE likely had a positive effect on hydroxyproline, MDA, and MPO. Fibrosis increased in the 14- and 21-day CAPE groups as compared with that in the control group, but the finding was not statistically significant. By 14 and 21 days, wound healing had accelerated, at which point fibrous tissue formation was undesirable. Based on these results, CAPE appeared to have positive effects on the early stage of wound healing by enhancing the wound healing rate. These effects can be attributed to the antimicrobial and antiinflammatory properties of CAPE.

Lopes-Rocha et al. reported that propolis had a beneficial effect on surgical wound healing in the oral cavity. In their study, CAPE reduced inflammation and granulation and accelerated the formation of tissue formation and epithelization [27]. In the present study, CAPE increased epithelium regeneration and had positive effects on wound healing on day 7, and it decreased epithelial regeneration on days 14 and 21. These findings indicate that these positive effects of CAPE decreased in the late period of wound healing. Furthermore, CAPE had a positive effect on the wound healing mechanism. 

Getting et al. found that TSG-6 reduced the level of inflammatory mediators: Tumour necrosis factor-alpha (TNF-α) and prostaglandin-E2 (PGE2) [28]. Beltran et al. suggested that after an intragingival injection of TSG-6, infiltration of neutrophils and proinflammatory cytokines as well as signs of inflammation decreased at 2 weeks [29]. In a study on rats in which corneal mechanical injury was induced, a TSG-6 injection into the anterior cavities of the rats’ eyes reduced corneal infection and inflammation [30]. In the current study, TSG-6 expression was low in the ABS group and control group but markedly increased in the CAPE group. These findings show that both CAPE and ABS had positive impacts on wound healing. The latter is likely to be due to the antiinflammatory and antimicrobial properties of CAPE and ABS.

According to one study, VEGF was particularly effective in terms of angiogenesis, epithelization, and collagen accumulation [31]. In the current study, VEGF expression was detected in the 7-day ABS group. This finding showed that ABS had a positive effect on wound healing by increasing vascularization. In the present study, VEGF was not expressed in the control group on day 21, whereas it was expressed on day 21 in the ABS group. VEGF expression increased in the 21-day CAPE group. 

In conclusion, in this study, the effects of ABS and CAPE on secondary healing of experimentally generated excisional wounds in the palatal mucosa of rats were evaluated histologically and biochemically. ABS was successful as a local haemostatic agent. CAPE was a biocompatible agent with oral mucosal tissues. ABS and CAPE appear to have a positive effect on reepithelization in the early stage of wound healing. ABS and CAPE may be beneficial in wound healing in periodontal surgery. Studies on postoperative and long-term results following the application of ABS and CAPE are needed to shed light on their clinical effects on wound healing in mucosal healing. 

## Acknowledgments

This study was supported by the Dicle University Scientific Research Projects Coordinator (project number: DİS.15.011).
